# Dietary Guanidine Acetic Acid Improves Ruminal Antioxidant Capacity and Alters Rumen Fermentation and Microflora in Rapid-Growing Lambs

**DOI:** 10.3390/antiox12030772

**Published:** 2023-03-22

**Authors:** Wenjuan Li, Zhaoyang Cui, Yaowen Jiang, Ailiyasi Aisikaer, Qichao Wu, Fang Zhang, Weikang Wang, Yukun Bo, Hongjian Yang

**Affiliations:** 1 State Key Laboratory of Animal Nutrition, College of Animal Science and Technology, China Agricultural University, Beijing 100193, China; 2 Zhangjiakou Animal Husbandry Technology Promotion Institution, Zhangjiakou 075000, China

**Keywords:** guanidine acetic acid, forage type, rumen fermentation, rumen microbiota, antioxidant capacity

## Abstract

Guanidine acetic acid (GAA) has been reported to improve growth performance, nutrient utilization, and meat quality in livestock. This study aimed to investigate whether coated GAA (CGAA) in comparison with uncoated GAA (UGAA) could have different effects on rumen fermentation, antioxidant capacity, and microflora composition in the rumen. Seventy-two lambs were randomly arranged in a 2 × 3 factorial experiment design with two diets of different forage type (OH: oaten hay; OHWS: oaten hay plus wheat silage) and three GAA treatments within each diet (control, diet without GAA addition; UGAA, uncoated GAA; CGAA, coated GAA). The whole feeding trial lasted for 120 days. The lambs in the OH group presented lower total volatile fatty acid (VFA), alpha diversity, *Firmicutes*, *NK4A214_group*, and *Lachnospiraceae_NK3A20_group* than those on the OHWS diet in the last 60 days of the feeding stage (*p* < 0.05). Regardless of what GAA form was added, dietary GAA supplementation increased the total VFA, microbial crude protein (MCP), adenosine triphosphate (ATP), and antioxidant capacity in rumen during lamb feedlotting (*p* < 0.05). However, molar propionate proportion, acetate:propionate ratio (A:P), and relative *Succiniclasticum* abundance decreased with GAA addition in the first 60 days of the growing stage, while the molar butyrate proportion and *NK4A214_group* (*p* < 0.05) in response to GAA addition increased in the last 60 days of feeding. These findings indicated that dietary GAA enhanced antioxidant capacity and fermentation characteristics in the rumen, but the addition of uncoated GAA in diets might cause some dysbacteriosis of the rumen microbiota.

## 1. Introduction

Guanidine acetic acid (GAA), with a molecular formula of C_3_H_7_N_3_O_2_ and a molecular weight of 117.11, is synthesized in the kidney, liver, and pancreas from *L*-arginine and glycine, and then converted to creatine to participate in the metabolism of energy and proteins [1]. As a nutritive feed additive, GAA has been used to improve growth performance, carcass characteristics, and meat quality in pigs [2], chickens [3], bulls [4], and sheep [5]. Unlike monogastric animals, host ruminants and rumen microorganisms are mutually beneficial and symbiotic [6]. Rumen microbial fermentation of feeds produces volatile fatty acids (VFA) and microbial protein to provide most of the available energy and protein required by host ruminants [7]. A previous study in Angus bulls noted that dietary GAA addition shifted ruminal fermentation towards greater propionate production [4]. The addition of GAA increased rumen total VFA production and microbial populations [8], with the ruminal degradation rate of GAA reported to be 47–49% in cattle [9]. However, it is unclear whether the coated GAA could sacrifice the aforementioned effects on rumen fermentation.

Oxidative stress in the rumen is detrimental to ruminant health [10]. Dietary additives with antioxidant features are often applied to avoid oxidative stress, especially during high-concentrate feeding [11]. Common antioxidant supplements include probiotic-based [12], selenium [13], and vitamin E [14]. As a nutritive additive, GAA donates an electron from its conjugate base and generates superoxide, a strong free radical [15], and may therefore be a direct pro-oxidant. However, GAA metabolites (e.g., creatine and arginine) might be able to quench free radicals after GAA ingestion [16,17,18]. A previous study on growing lambs reported that dietary GAA addition elevated the activities of serum catalase (CAT) and glutathione peroxidase (GSH-Px) and decreased malondialdehyde (MDA) content in skeletal muscle tissue [5]. However, it is not clear whether dietary GAA addition could present antioxidant capacity in the rumen, or what difference there could be compared with the coated GAA.

It is well known that forage type is an important factor affecting rumen fermentation as growth performance in sheep feeding practice. For instance, feeding sheep oaten hay as a forage source was found to maintain the stable state of their rumen internal environment and the growth of rumen microorganisms [19]. Wheat silage (WS) can provide an interim forage during the period when the previous year’s hay or silage has run out and the present year’s has not been harvested. A previous study in finishing beef cattle noted that feeding WS was found to decrease the acetate:propionate ratio in rumen and presented greater growth performance [20]. However, relevant research on sheep is scarce. Considering sheep are less tolerant to acid whole silage as sole forage, lamb diets with WS plus OH in comparison with sole OH as forage type were applied in the present lamb feedlotting trial.

The primary objective was to elucidate whether or not the coated GAA in comparison with uncoated GAA addition could improve rumen fermentation, antioxidant capacity, and how they affect rumen microflora in rapid-growing lambs, depending on the diet with different forage type sources.

## 2. Materials and Methods

### 2.1. Animal Ethics Statement

The experimental animals, design, and animal management in the present study followed the Guidelines of the Beijing Municipal Council on Animal Care (with protocol CAU20171014-1) and were in accordance with the recommendations of the academy’s guidelines for animal research.

### 2.2. Guanidinoacetic Acid Products

The uncoated GAA (available content of 984 g/kg, the average rumen degradation rate of UGAA is 50.9%) and coated GAA (available content of 600 g/kg, the packaging material was mainly fat powder, the average rumen degradation rate of CGAA is 15.8%). added in this study were in powder form and provided by Hebei Guang rui Company (Shijiazhuang, China).

### 2.3. Experimental Animals, Diets, and Design

Seventy-two two-month-old healthy male small-tailed Chinese Han lambs (a breed of sheep native to Shandong Province of China, with a rate of reproduction reaching 229%) initially weighing 12 ± 1.6 kg in body weight (BW) were chosen as experimental animals and fed total mixed rations (TMR). A 2 × 3 factorial feeding experimental design was applied to divide the animals into two forage types of TMRs (OH: oaten hay; OHWS: oaten hay plus wheat silage), and three GAA addition groups (GAA: 0 g/kg; UGAA: uncoated GAA, 1 g/kg; CGAA: coated GAA, 1 g/kg). GAA (UGAA and CGAA) was added to the concentrates, then mixed with the corresponding forage, and divided into two daily feeds (08:00 and 16:00). Each treatment was randomly divided into four bamboo-slotted bedding pens, and each pen was arranged with three lambs, while clean, fresh water was available at all times. All rations were formulated to satisfy the nutrient requirement of 300 g gain/day [21]. The composition and nutrient levels of the experimental diet are shown in Table A1. The study periods consisted of 7 days of adaptation and 120 days of a 2-stage data collection.

### 2.4. Sample Collection and Analyses

#### 2.4.1. Rumen Fluid Sampling

Rumen fluid samples were collected at the end of every stage (d 60 and d 120). One hour after the morning meal, an oral stomach cannula (MDW15, Colebo Equipment Co., Ltd., Wuhan, China) and a 200 mL syringe were used to collect rumen fluid samples. The first 2 tubes of rumen fluid were discarded to avoid saliva contamination [8], and a 100 mL rumen fluid sample was collected from 8 lambs in each group. Ruminal pH was immediately determined by a digital pH meter (Testo205 type, Testo AG, Lenzkirch, Germany). Subsequently, the samples were strained through four layers of cheesecloth. A total of 0.25 mL of metaphosphoric acid (25 g/100 mL) was added to aliquots of 1 mL rumen fluid, which were centrifuged at 20,000× *g* at 4 °C for 15 min to determine the VFA, and two aliquots of 2 mL samples were taken to determine ammonia nitrogen (NH_3_-N) concentration and microbial protein (MCP). Three aliquots of 1 mL samples were taken to determine GAA, creatine, guanidinoacetate N-methyltransferase (GAMT), *L*-Arginine: glycine amidino-transferase catalyzes (AGAT), adenosine triphosphate (ATP), superoxide dismutase (SOD), catalase (CAT), gluta-thione peroxidase (GSH-Px), malondialdehyde (MDA), total antioxidant capacity (T-AOC), glutathione (GSH), and microbiota.

#### 2.4.2. Rumen Fermentation Index Measurement

The centrifuged sample referred to above was filtered using a 0.22 mm syringe filter. The VFA was quantified using a high-performance gas chromatograph (HPGC; GC-128; INESA Corporation) equipped with a hydrogen flame detector and a capillary column (FFAP, Zhonghuida Instruments Co., Ltd., Dalian, China; 50 m long, 0.32 mm diameter, 0.50 µm film). The VFA was identified and quantified from the chromatograph peak areas using calibration with external standards [22]. The rumen liquid NH_3_-N was measured according to Bremner and Keeney’s (1965) method [23], which calls for the use of a spectrophotometer (UV-6100, Mapada Instruments Co., Ltd., Shanghai, China). The microbial protein was then quantified using the purine derivative method [24].

#### 2.4.3. Ruminal GAA, Creatine, Enzyme Activity, and Antioxidant Capacity Related to GAA Metabolism

GAA and creatine in rumen liquid were determined reference to the study by Wada et al. [25]. To this end, 1 mL of rumen liquid was aliquoted into another centrifuge tube, and 3 mL 5% aqueous solution of sodium sulfosalicylate was added and mixed into the rumen liquid to precipitate the protein. The mixture was incubated at room temperature for 10 min and then centrifuged at 12,000× *g* for 10 min. Subsequently, the centrifuged sample mentioned above was filtered using a 0.22 mm syringe filter for the determination. The determination conditions were as follows: C18 weak acid cation exchange column (4.6 mm × 250 mm, 5 μm) was used; the flow rate was 0.6 mL/min; the column temperature was 30 °C; the detection wavelength was 210 nm; the elution mode was one-time linear elution; and the sample size was 10 μL.

Thiobarbituric acid reactive substances (TBARS) assay measured MDA as a product of lipid peroxidation. The activity of enzymes SOD, CAT, and GSH-PX, and the contents of T-AOC, GSH, and ATP in rumen were measured using an assay kit (Jiancheng Biochemical Reagent Co., Nanjing, China) according to the manufacturer’s instructions. The GAMT and AGAT activities were measured in ruminal fluid of the lambs using a corresponding enzyme-linked immunosorbent assay kit (JinHaiKeYu Biochemical Reagent Co., Beijing, China).

#### 2.4.4. Ruminal Microorganism DNA Extraction, PCR Amplification, and Sequencing

A Fast DNA^®^ soil DNA Kit (MP Biomedicals, Santa Ana, CA, USA) was used to extract the total microbial DNA from 60 rumen fluid samples according to the manufacturer’s instructions. DNA purity and concentration were detected with a NanoDrop2000 UV-vis spectrophotometer (Thermo Scientific, Wilmington, DE, USA), and DNA integrity was assessed with 1% agarose gel electrophoresis. The following amplification primers for 16 rRNA (V3 + V4) were used: 341F: 5′CCTAYGGGGBGCASCAG3′; 806R: 3′GGACTACNNGGGTATCTAAT5′. The PCR amplification of the 16S rRNA gene is shown in Table A2, and the PCR system is shown in Table A3. The PCR product was extracted from 2% agarose gel and purified using an AxyPrep DNA Gel Extraction Kit (Axygen Biosciences, Union City, CA, USA) according to the manufacturer’s instructions and quantified using a Quantus™ Fluorometer (Promega, Madison, WI, USA). Sequencing was conducted on a MiSeq PE300 platform (Illumina, San Diego, CA, USA) according to the standard protocols of Majorbio Bio-Pharm Technology Co., Ltd. (Shanghai, China). The raw reads were deposited into the NCBI Sequence Read Archive (SRA) database (PRJNA846357).

#### 2.4.5. Processing of Sequencing Data

Raw sequencing data were merged using the sliding window method and paired using FLASH (version 1.2.11, https://ccb.jhu.edu/software/FLASH/index.shtml (accessed on 27 September 2021)) on the basis of overlapping bases. The allowed mismatches of barcode and primer mismatch were 0 and 2, respectively. UPARSE software (version 7.0.1090, http://drive5.com/uparse/ (accessed on 9 October 2021)) was used to perform operational taxonomic unit (OTU) sequence clustering, and their relative abundances were used to calculate rarefaction curves and values of the Shannon Diversity Index using UPARSE version 7.1 (http://drive5.com/uparse/ (accessed on 8 November 2021)). The ribosomal database project (RDP) classifier (http://rdp.cme.msu.edu/ (accessed on 8 November 2021)) was used to classify and annotate each sequence through comparison to Silva (Release132, http://www.arb-silva.de/ (accessed on 8 November 2021)) with a comparison threshold of 70% [26]. Alpha diversity analysis that included the Sobs, Shannon, Ace and Coverage indices at the OTU level was conducted using Mothur software (version 1.30.2, https://www.mothur.org/wiki/Download_mothur (accessed on 8 November 2021)). The beta diversity analysis and the principal coordinates analysis (PCoA) was analyzed at the OTU level with the distance algorithm of weighted normalized UniFrac. The differential bacteria were analyzed using the linear discriminant analysis effect size (LEfSe) software (http://huttenhower.sph.harvard.edu/galaxy/root?Tool_id¼lefse_Upload (accessed on 8 November 2021)). Bar plots and non-metric multidimensional scaling (NMDS) were generated using R software.

### 2.5. Statistical Analysis

Data for each feeding stage were analyzed using the MIXED procedure of the Statistical Analysis System Institute [27]. Ruminal antioxidant capacity, rumen fermentation, and GAA metabolism were analyzed. The model was applied as follows:

$$Y_{ijk}=\mu +G_{i}+F_{j}+{\left(G\times F\right)}_{ij}+R_{k}+eijk$$

where Y_ijk_ is the dependent variable, µ is the overall mean, G_i_ is the fixed effect of GAA products (i = 3: control, uncoated GAA, coated GAA), F_j_ is the fixed effect of total mixed ration type with different forage types (OH and OHWS), and G × F is the interaction of GAA and ration type. R_k_ is the random effect of animals (k = 12 per treatment) or pens (k = 3 per treatment), and eijk is the residual error term. The least squares means and standard errors of the means were calculated using the LSMEANS statement of the SAS software. Significance was declared at *p* ≤ 0.05, unless otherwise noted.

## 3. Results

### 3.1. Rumen Fermentation

At stage 1, interaction between forage type and GAA addition was found for the total VFA, acetate, and A:P (Table 1). UGAA and CGAA addition in the OH group increased the total VFA (*p* < 0.05), whereas only CGAA addition increased the total VFA in OHWS group (*p* < 0.05). The UGAA supplementation in the OH group increased the acetate proportion and decreased the A:P (*p* < 0.05), whereas there was no difference in the OHWS group (*p* > 0.05). Similarly, no difference was observed for rumen fermentation parameters with the two forage types (*p* > 0.05). Both UGAA and CGAA increased the content of total VFA (*p* < 0.001), A:P (*p* = 0.011), NH_3_-N (*p* = 0.025), and MCP (*p* = 0.015), but decreased the propionate proportion (*p* = 0.009) and pH value (*p* = 0.023). The addition of CGAA to the OH diet increased the butyrate proportion compared to the UGAA and control (*p* < 0.05).

At stage 2, the significant Forage × GAA interaction was observed on total VFA, acetate, and A:P. Dietary UGAA and CGAA addition in the OH group increased the total VFA (*p* < 0.05) and A:P (*p* < 0.05), but this phenomenon did not occur in the OHWS group. However, UGAA and CGAA decreased the acetate proportion in the OHWS group, and there was no change in the OH group. Compared to the OHWS diet, the lambs fed the OH diet had lower total VFA (*p* = 0.018). The concentration of total VFA (*p* < 0.001) and percentage of butyrate (*p* = 0.008) and MCP (*p* = 0.011) were higher with the addition of UGAA or CGAA, and the pH value showed a tendency to decrease (*p* = 0.082). The other indicators did not alter in response to the addition of GAA.

### 3.2. Ruminal GAA, Creatine, ATP, and Related Metabolic Enzymes

As shown in Table 2, the forage × GAA interaction was not significant for GAA, creatine, ATP, and related metabolic enzymes at stage 1 and stage 2.

At stage 1, no difference was observed for ruminal GAA, creatine, GAMT and AGAT in the two forage types (*p* > 0.05). UGAA or CGAA addition increased GAA (*p* < 0.001), GAMT (*p* = 0.002), and ATP (*p* = 0.014) but decreased AGAT (*p* = 0.025) activity compared to the control.

At stage 2, the concentration of GAA, creatine, ATP, and the activity of GAMT and AGAT did not differ between the two forage types (*p* > 0.05). Furthermore, regardless of the form of GAA added, the lambs presented greater rumen GAA (*p* < 0.001) and ATP (*p* = 0.001) than the control. No significant differences were observed among the treatments in the rumen creatine, or in the activity of GAMT and AGAT (*p* > 0.05).

### 3.3. Ruminal Microbiota

The alpha diversity was not affected by forage type or GAA addition, except that the lambs in the OH group, in comparison with the OHWS group, presented lower sobs (*p* = 0.017), ace (*p* = 0.011), and chao (*p* = 0.010) in stage 2 (Supplementary Table S1).

In stage 1, the effects of the interaction between forage type and GAA addition were found on the *Bacteroidota* (Table 3). Dietary CGAA in the OH group presented greater *Bacteroidota* than dietary CGAA (*p* < 0.05), whereas there was no difference in the OHWS group (*p* > 0.05). The relative abundance of *Firmicutes*, *Bacteroidota*, *Synergistota*, *Patescibacteria*, and *Proteobacteria* did not alter in response to either forage type or GAA addition.

In stage 2, the effects of the interaction between forage type and GAA addition were found on the *Firmicutes* and *Bacteroidota*. Dietary UGAA and CGAA in the OH group presented greater *Firmicutes* compared with the control group (*p* < 0.05); dietary UGAA in the OH group presented lower *Bacteroidota* compared with the control group (*p* < 0.05), whereas there was no difference in the OHWS group (*p* > 0.05). Compared with the OHWS diet, the lambs fed the OH diet had lower (*p* = 0.039) *Firmicutes*. The relative abundance of microbiota did not alter in response to GAA addition (*p* > 0.05). It is worth mentioning that whatever form of GAA was added, the lambs fed the OHWS diet presented lower *Patescibacteria* levels (*p* < 0.05).

At the genus level (Table 4), forage type and GAA addition interacted (*p* = 0.040) to affect the abundance of the *NK4A214_group* at stage 1. Dietary UGAA in the OH group presented a lower *NK4A214_group* compared with the control group (*p* < 0.05), whereas there was no difference in the OHWS group (*p* > 0.05). The forage type did not affect the microbial composition at the genus level (*p* > 0.05). Regardless of the form of GAA added, the population of *Succiniclasticum* decreased (*p* = 0.049) compared to the control. Furthermore, dietary CGAA in the OH group presented higher (*p* < 0.05) *Prevotella* abundance compared with dietary CGAA and the control. The relative abundances of *norank_f_norank_o_Clostridia_UCG-014* decreased (*p* < 0.05) with UGAA addition in the OH group compared to the control.

Interaction between forage type and GAA addition affected the abundance of *Prevotella* (*p* = 0.042), *Ruminococcus* (*p* = 0.027), and *Succiniclasticum* (*p* = 0.006) at stage 2; the relative abundances of *Prevotella* decreased with UGAA and CGAA addition (*p* < 0.05); the relative abundances of *Succiniclasticum* decreased and *NK3A20* increased with UGAA addition compared to the control in the OH group (*p* < 0.05), whereas there was no difference in the OHWS group (*p* > 0.05). However, the abundances of *Ruminococcus* decreased with the addition of UGAA compared to the control and *NK4A214_group* increased with the UGAA and CGAA addition in OHWS group (*p* < 0.05), whereas there was no difference in the OH group (*p* > 0.05). The lambs in the OH group in comparison with the OHWS group presented lower *NK4A214_group* (*p* = 0.008) and *NK3A20* (*p* = 0.038). Both forms of GAA resulted in greater *NK4A214_group* abundances (*p* = 0.040). The other genera did not alter in response to either forage type or GAA addition (*p* > 0.05).

**Figure 1:**
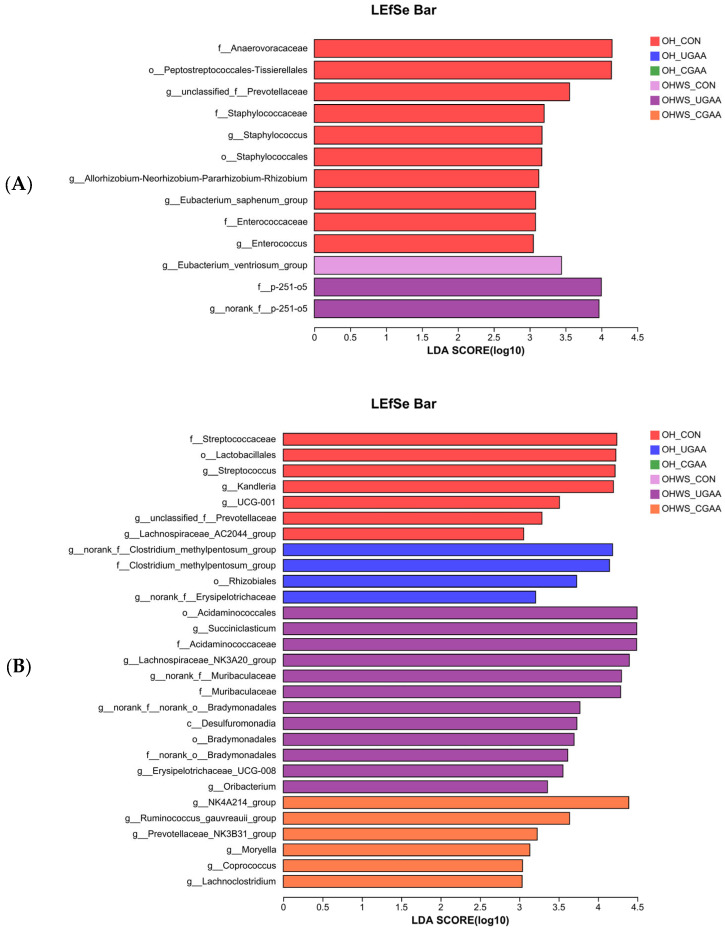
LEfSe bar showing the taxonomic differences in the rumen of lambs in stage 1 (**A**) and stage 2 (**B**). All identified taxonomy was significantly different based on LDA score (**B**) larger than 3.0.

At stage 1, 13 bacterial taxa were identified by LEfSe as significantly enriched in the rumen, comprising 10 (*f_Anaerovoracaceae*, *o_Peptostreptococcales-Tissierellales*, *g_unclassified_f_Prevotellaceae*, *f_Staphylococcaceae*, *g_Staphylococcus*, *o_Staphylococcales*, *g_Allorhizobium-Neorhizobium-Pararhizobium-Rhizobium*, *g_Eubacterium_saphenum_group*, *f_Enterococcaceae*, and *g_Enterococcus*) in the control group with the OH diet, and 1 (*g_Eubacterium_ventriosum_group)* and 2 (*g_norank_f_p-251-o5, f_p-251-o5*) in the control and UGAA addition with the OHWS diet, respectively (Figure 1A).

At stage 2, 29 bacterial taxa were identified by LEfSe as significantly enriched in the rumen, comprising 7 (*f_Streptococcaceae*, *o_Lactobacillales*, *g_Streptococcus*, *g_Kandleria*, *g_UCG-001*, *g_unclassified_f_Prevotellaceae*, and *g_Lachnospiraceae_AC2044_group*) and 4 (*g_norank_f_Clostridium_methylpentosum_group*, *f_Clostridium_methylpentosum_group*, *o_Rhizobiales*, and *g_norank_f_Erysipelotrichaceae*) in the control and with UGAA addition in the OH group, respectively, and 12 (*o_Acidaminococcales*, *g_Succiniclasticum*, *f_Acidaminococcaceae*, *g_Lachnospiraceae_NK3A20_group*, *g_norank_f_Muribaculaceae*, *f_Muribaculaceae*, *g_norank_f_norank_o_Bradymonadales*, *c_Desulfuromonadia*, *o_Bradymonadales*, *f_norank_o_Bradymonadales*, *g_Erysipelotrichaceae_UCG008*, *g_Oribacterium*) and 6 ((*g_NK4A214*, *g_Ruminococcus_gauvreauii_group*, *g_Prevotellaceae_NK3B31_group*, *g_Moryella*, *g_Coprococcus*, *g_Lachnoclostridium*) in the control and with CGAA addition in the OHWS group, respectively (Figure 1B).

### 3.4. Rumen Antioxidant Capacity

As shown in Table 5, at stage 1, the Forage × GAA interaction effect was exerted on the SOD, CAT, and GSH-Px activities but not on the levels of T-AOC, GSH, and MDA. For the OH diet, the highest T-AOC content and SOD and CAT activities were observed with the CGAA addition, whereas for the OHWS diet, UGAA addition resulted in the maximum amount of T-AOC content and SOD and CAT activities. The forage type did not affect antioxidant capacity. GAA (UGAA and CGAA) supplementation in the rumen increased the T-AOC (*p* < 0.001), SOD (*p* < 0.001), CAT (*p* < 0.001), GSH-Px (*p* < 0.001), and GSH (*p* < 0.001), whereas the level of MDA (*p* < 0.001) decreased with the addition of GAA.

At stage 2, an interaction effect was observed in the T-AOC, MDA levels and SOD, and GSH-Px activities. CGAA addition had the highest SOD activity, GSH-Px activity, and T-AOC content, and the lowest MDA content in the OH diet, but in the OHWS diet, UGAA addition achieved similar results. The type of forage did not affect the antioxidant capacity. Moreover, the T-AOC (*p* < 0.001), SOD (*p* < 0.001), CAT (*p* = 0.048), GSH-Px (*p* < 0.001), and GSH (*p* = 0.009) increased but the level of MDA (*p* < 0.001) decreased with GAA addition.

## 4. Discussion

Oxidative stress is a dysregulation between the production of reactive oxygen species and the endogenous antioxidant defense mechanisms [28]. For ruminants, high-concentrate diets have been widely associated with oxidative stress by increasing the metabolic rate [29]. In general, SOD, CAT, and GSH-Px activity, T-AOC, GSH, and MDA contents in serum are usually measured to reflect whether or not oxidative stress has occurred in the body. However, it is unknown whether oxidative stress could be reflected by measuring the aforementioned indices in rumen liquid. In a previous study, both CAT and GPx4 activities were elevated and the MDA content was decreased in serum when dietary GAA addition was applied in growing lambs [5]. In a subsequent study with Holstein dairy cows, dietary GAA addition was found to promote the growth of rumen microorganisms [30]. However, it is not clear whether the above responses to GAA could be associated with the rumen fermentation characteristics and microbial stability of the environment inside the rumen and how their relationship with antioxidant capacity is directly measured in the rumen fluids.

### 4.1. Rumen Fermentation

Total VFA concentration, pH value, NH_3_-N, and MCP levels are the main internal environmental indicators of rumen fermentation [8]. Among them, total VFA production was believed to account for over 70% of the energy requirement [31]. In one of our previous studies with growing lambs, increasing the foxtail millet silage replacement of peanut vine hay in rations exhibited a lower pH value and greater total VFA production [32]. In the present study, regardless of what form of GAA was applied, the lambs in the OHWS group in comparison with those in the OH group presented greater total VFA production during the last 60 days of the feeding stage, suggesting that WS in comparison with OH might exhibit rumen digestibility.

In one of our previous studies, the application of GAA was confirmed to improve growth performance and apparent total tract nutrient digestibility in lamb feeding practice [33]. In the present study, GAA feeding resulted in higher total VFA assessed at two stages. These results could be partly explained by the stimulation of nutrient degradation with dietary GAA supplementation in the rumen. Similarly, the addition of GAA has also been shown to increase rumen total VFA production in Holstein dairy cows [30]. The decrease in ruminal pH was commonly believed to be associated with an increase in the total VFA concentration. In the present study, the relatively low ruminal pH of 6.74 was observed for UGAA added to the OHWS diet group, and this pH was not low enough to have a negative impact on ruminal microorganisms [34]. Previous studies have reported that addition of GAA (0.6 or 0.9 g/kg basal diet) increased molar propionate proportion and A:P in Holstein dairy cows [30]. This phenomenon was not observed in the present study, and was probably a result of the differences in lamb species, ages, and basal diets. Researchers have also indicated that GAA can be used by microbes as an N source to synthesize their proteins [4], as observed in the present study in two stages.

### 4.2. Ruminal GAA, Creatine, ATP, and Related Metabolic Enzymes

In the present study, rumen GAA, creatine, and ATP levels, as well as GAMT and AGAT activities, did not vary with forages. This was probably due to the same ratio of concentrate to forage in both TMRs, as replacing 36% OH with WS had little effect on indicators related to energy metabolism in the rumen.

A previous study noted that GAA in the diet was absorbed by the gut through the portal blood into the liver for creatine synthesis [35], and GAMT and AGAT were key enzymes in this process [36]. Decreased rumen AGAT activity was observed with the addition of GAA at stage 1 in the present study. This was consistent with the finding that AGAT can be feedback-inhibited by GAA [37]. Most metabolic studies on GAA have focused on the liver, blood, urea, and small intestinal segment mucosa [8,36,38]. The addition of GAA increased the rumen GAA content at both stages 1 and 2, confirming that GAA was degraded in the rumen and significantly correlated with the degradation rate (UGAA, 50.9%; CGAA, 15.8%). Furthermore, ATP level improved with GAA addition compared to the control. It also supports the theory that GAA can provide energy to microbes [4]. Interestingly, we did not observe a difference in ATP content between the two forms of GAA, suggesting that not all of the GAA degradation was used to provide energy, and would even be a waste, thus necessitating coating.

### 4.3. Ruminal Microbiota

The alpha diversity analysis of lamb rumen flora in our experiment showed that the coverage of each group was higher than 99% in two stages, indicating that the sequencing results truly reflected the species and structural diversity of the lamb rumen bacterial community. Forages did not affect alpha diversity in stage 1, which is consistent with previous research [39]. However, compared with the OHWS group, the sobs, ace, and chao index decreased with the OH diet in stage 2. These results indicate that rumen microbial richness varies with diet and host [40]. In the present study, no difference emerged in the alpha diversity with the addition of GAA, indicating that GAA did not affect the richness or evenness of lamb rumen microorganisms.

In this study, at the phylum level, *Firmicutes* and *Bacteroidota* were the dominant phyla, which are known to be involved in carbohydrate and protein degradation [33]. The interaction between forage type and GAA addition was found to affect the abundance of *Bacteroidota*. We suspected that suitable GAA (CGAA) could promote *Bacteroidota* enrichment in the rumen, and a high concentration (UGAA) would affect the abundance of *Bacteroidota.* At stage 2, compared with the OHWS diet, the lambs fed the OH diet had a lower relative abundance of *Firmicutes*, which represented the predominant bacterium within the rumen, mainly comprising diverse fibrolytic and cellulolytic bacterial genera [41]. Similar to stage 1, dietary UGAA in the OH group presented a lower relative abundance of *Bacteroidota,* compared with the control group, which contributed to the release of energy from dietary fiber and starch [42].

At the genus level, *Prevotella* participates in the hydrolysis of proteins and the absorption of peptides in the rumen [43] and propionate production [44]. *Prevotella* enrichment helps to increase the antioxidant capacity in rumen fluid [45]. In the present study, the CGAA addition exhibited a higher relative abundance of *Prevotella* than the UGAA addition and the control with the OH diet. This corresponds to the highest propionate proportion. This also confirms that the addition of GAA can improve antioxidant capacity in rumen fluid. Furthermore, the addition of UGAA decreased the abundance of *NK4A214_group, Succiniclasticum*, and *Clostridia_UCG-014* in the OH diet at stage 1. This suggests that the degradation of GAA in the rumen may lead to partial microbiota disorders. The same phenomenon was manifested in *Ruminococcus* and *Succiniclasticum* at stage 2. Higher relative abundances of *NK4A214_group* and *NK3A20* were observed with the OHWS diet at stage 2. This may be because whole wheat silage contains higher starch than oaten hay. Zhang et al. [46] also found that *NK4A214_group* and *NK3A20* enhanced starch and sucrose metabolism. Furthermore, *NK4A214_group* and *NK3A20* are butyric acid-producing bacterium [47,48], thus explaining the corresponding increase in butyric acid with the addition of GAA.

Apart from *norank_f_p-251-o5* and *f_p-251-o5* in the UGAA with the OHWS diet, no differential bacteria were identified in stage 1 by LEfSe analysis. However, at stage 2, the OH diet with the UGAA group was enriched with *norank_f_Clostridium_methylpentosum_group*, *f_Clostridium_methylpentosum_group*, *o_Rhizobiales*, and *norank_f_Erysipelotrichaceae*. Other researchers have reported that *Clostridium_methylpentosum* is a ring-shaped intestinal bacterium that ferments only methylpentoses and pentoses [49]. The *norank_f_Erysipelotrichaceae* was reported to relate to metabolic disorder and inflammation-related gastrointestinal diseases [50]. This indicates that the addition of CGAA may lead to disorder of the internal environment and even inflammatory reaction in rumen. In the present study, the *NK4A214_group*, *Ruminococcus_gauvreauii_group*, *Prevotellaceae_NK3B31_group*, *Moryella*, *Coprococcus*, and *Lachnoclostridium* were enriched in the group fed with the OHWS and CGAA diet. Previous research has shown that *Moryella*, *Coprococcus,* and *Lachnoclostridium* are butyric acid-producing bacteria [51,52]. Previous studies have shown that butyrate treatment of the colon increases glutathione content [53]. The increase in butyric acid-producing bacteria may also provide indirect evidence that GAA improves antioxidant capacity in this study.

### 4.4. Rumen Antioxidant Capacity

A high concentrate diet in ruminants results in a massive release of bacterial endotoxins, which cause rumen epithelial cells to generate a certain amount of reactive oxygen species and lead to oxidative stress [54]. This phenomenon causes oxidative damage and decreases the activities of GSH-Px, CAT, and SOD [55]. SOD, CAT, and GSH-Px are intracellular antioxidant enzymes involved in the enzymatic antioxidant system against oxidative stress [56]. The SOD involved in the superoxide anion free radical (O_2_^−^) scavenging process in cells [57]. MDA is one of the meta-stable end products of lipid peroxidation [58]. Consistent with our hypothesis, the forage type did not affect antioxidant capacity due to the consistent forage-to-concentrate ratio. GAA has been reported to have a direct [59] or indirect antioxidant effect [60,61]. In our study, GAA addition increased SOD, CAT, and GSH-Px activities, as well as levels of T-AOC and GSH, but decreased MDA level at both stages 1 and 2, suggesting that GAA enhanced the antioxidant capacity in rumen. There are limited studies on the antioxidant properties of GAA in rumen fluid. We speculate that there are three possible reasons for this phenomenon. The content of creatine in rumen fluid increases numerically with the addition of GAA, as creatine has been proven to have the ability to remove O_2_- [60]. In addition, dietary GAA supplementation can save arginine, and *L*-Arginine alleviates oxidative stress by modulation of intestinal microbiota in intrauterine growth-retarded suckling lambs [61]. Another possible reason is that GAA increases the proportion of butyric acid in the rumen fluid, and butyrate improves the level of oxidative stress in intestinal mucosal cells [62]. It is interesting to find that OH diets had higher antioxidant capacity with CGAA addition, while OHWS diets had more significant antioxidant capacity with the addition of UGAA. This suggests that, in practice, we can choose the appropriate form of GAA according to the different types of forage.

Examining these findings together, the authors of the present study speculated that GAA supplementation could improve antioxidant capacity and rumen fermentation via providing energy for rumen microorganisms since partial GAA was metabolized. However, GAA degradation would also cause rumen dysbacteriosis as noted by the ruminal microbiota analysis. The results obtained in the present study suggested that the antioxidant capacity of GAA could be reflected by measuring the relevant indicators in rumen fluids samples; the increase in antioxidant capacity may be related to the enrichment of butyric acid-producing bacteria.

## 5. Conclusions

Our findings demonstrated that dietary GAA exhibited higher antioxidant capacity, total VFA, and microbial protein production in lambs fed with different forage types. The application of GAA as a feed additive has a bright application prospect of reducing oxidative stress and providing energy to support rumen fermentation in rapid-growing lambs. However, considering the rumen microbial stability, coated GAA is necessary to avoid rumen dysbacteriosis in feeding practice.

## Figures and Tables

**Table 1:** Effects of forage type and GAA addition on rumen fermentation in feedlotting lambs.

Items	Forage	GAA Addition			SEM	*p*-Value		
		Control	UGAA	CGAA		Forage	GAA	Forage × GAA
Stage 1 (60 d)								
Total VFA, mmol/L	OH	78.79 ^b^	84.20 ^a^	86.21 ^a^	0.78	0.245	<0.001	0.010
	OHWS	82.39 ^b^	83.79 ^ab^	85.27 ^a^				
Acetate, %	OH	56.11 ^b^	56.69 ^a^	56.17 ^b^	0.16	0.111	0.089	0.071
	OHWS	55.93	56.07	56.32				
Propionate, %	OH	32.28 ^a^	31.26 ^b^	32.43 ^a^	0.18	0.084	0.009	0.001
	OHWS	32.34	32.32	32.07				
Butyrate, %	OH	9.06 ^b^	9.09 ^b^	9.57 ^a^	0.16	0.508	0.257	0.132
	OHWS	9.12	9.21	9.14				
A:P	OH	1.74 ^b^	1.81 ^a^	1.73 ^b^	0.01	0.059	0.011	0.001
	OHWS	1.73	1.74	1.76				
pH	OH	6.86	6.77	6.80	0.03	0.240	0.023	0.917
	OHWS	6.82	6.74	6.78				
NH_3_-N, mg/dL	OH	9.51	9.89	9.83	0.16	0.592	0.025	0.873
	OHWS	9.39 ^b^	9.92 ^a^	9.70 ^ab^				
MCP, mg/mL	OH	42.51	43.24	43.25	0.33	0.394	0.015	0.914
	OHWS	42.58 ^b^	43.56 ^a^	43.56 ^a^				
Stage 2 (120 d)								
Total VFA, mmol/L	OH	83.11 ^b^	86.74 ^a^	87.42 ^a^	0.59	0.018	<0.001	0.007
	OHWS	86.70	86.93	87.54				
Acetate, %	OH	54.40	54.91	54.86	0.22	0.066	0.575	0.003
	OHWS	54.98 ^a^	54.07 ^b^	54.10 ^b^				
Propionate, %	OH	33.95 ^a^	32.95 ^b^	33.32 ^b^	0.24	0.817	0.133	0.067
	OHWS	33.34	33.37	33.63				
Butyrate, %	OH	8.94	9.29	9.48	0.19	0.334	0.008	0.787
	OHWS	8.97 ^b^	9.61 ^a^	9.58 ^a^				
A:P	OH	1.60 ^b^	1.67 ^a^	1.65 ^a^	0.01	0.311	0.416	0.003
	OHWS	1.65	1.62	1.61				
pH	OH	6.89 ^a^	6.69 ^b^	6.77 ^ab^	0.05	0.561	0.082	0.364
	OHWS	6.83	6.79	6.81				
NH_3_-N, mg/dL	OH	9.96	10.39	10.28	0.35	0.158	0.160	0.773
	OHWS	10.09	10.97	10.83				
MCP, mg/mL	OH	43.44	44.30	44.10	0.30	0.825	0.011	0.978
	OHWS	43.46 ^b^	44.43 ^a^	44.12 ^ab^				

OH, the rations with the forage type of oaten hay; OHWS, the rations with the forage type of oaten hay plus wheat silage; VFA, volatile fatty acids; A:P, the ratio of acetate to propionate; NH_3_-N, ammonia nitrogen; MCP, microbial crude protein; SEM, standard error of least squares means; ^a,b^ means with different superscripts were significantly different (*p* < 0.05).

**Table 2:** Effects of forage type and GAA addition on ruminal GAA, creatine, ATP, and related metabolic enzymes.

Items	Forage	GAA Addition			SEM	*p*-Value		
		Control	UGAA	CGAA		Forage	GAA	Forage × GAA
Stage 1 (60 d)								
GAA, μg/mL	OH	57.56 ^c^	99.90 ^a^	86.45 ^b^	1.97	0.899	<0.001	0.983
	OHWS	57.87 ^c^	99.70 ^a^	86.97 ^b^				
Creatine, μg/mL	OH	34.80	35.18	36.83	1.17	0.099	0.341	0.634
	OHWS	36.12	38.01	37.49				
GAMT, U/L	OH	7.96 ^b^	9.65 ^a^	9.58 ^a^	0.40	0.973	<0.001	0.873
	OHWS	7.97 ^b^	9.87 ^a^	9.38 ^ab^				
AGAT, U/L	OH	13.56 ^a^	13.30 ^a^	12.68 ^b^	0.31	0.973	0.025	0.998
	OHWS	13.55 ^a^	13.31 ^ab^	12.65 ^b^				
ATP, μmol/L	OH	3.61	3.66	3.64	0.02	0.571	0.014	0.725
	OHWS	3.60 ^b^	3.65 ^a^	3.65 ^a^				
Stage 2 (120 d)								
GAA, μg/mL	OH	62.34 ^c^	100.03 ^a^	87.15 ^b^	1.59	0.856	<0.001	0.838
	OHWS	61.24 ^c^	99.64 ^a^	87.93 ^b^				
Creatine, μg/mL	OH	36.64	37.69	37.60	1.41	0.205	0.724	0.949
	OHWS	38.29	39.51	38.57				
GAMT, U/L	OH	11.08	11.13	11.57	0.24	0.943	0.430	0.594
	OHWS	11.18	11.33	11.31				
AGAT, U/L	OH	14.13	14.54	14.70	0.45	0.429	0.157	0.870
	OHWS	14.27	14.65	15.16				
ATP, μmol/L	OH	3.62 ^b^	3.66 ^ab^	3.69 ^a^	0.02	0.388	0.001	0.937
	OHWS	3.61 ^b^	3.65 ^ab^	3.67 ^a^				

OH, the rations with the forage type of oaten hay; OHWS, the rations with the forage type of oaten hay plus wheat silage; GAMT, guanidinoacetate N-methyltransferase; AGAT, *L*-Arginine: glycine amidine transferase catalyzes; ATP, adenosine triphosphate; SEM, standard error of the difference of the means; ^a,b,c^ means with different superscripts were significantly different (*p* < 0.05).

**Table 3:** Microbial community analysis at the phylum level (relative abundance > 1%) of microbiomes obtained from rumen fluids of lambs at different feeding stages.

Items	Forage	GAA Addition			SEM	*p*-Value		
		Control	UGAA	CGAA		Forage	GAA	Forage × GAA
Stage 1 (60 d)								
*Firmicutes*	OH	61.91	59.20	52.22	6.07	0.112	0.612	0.297
	OHWS	68.25	58.79	71.10				
*Bacteroidota*	OH	28.11 ^ab^	19.23 ^b^	41.50 ^a^	5.31	0.747	0.447	0.026
	OHWS	26.56	33.82	24.21				
*Synergistota*	OH	0.28	14.93	0.48	5.25	0.277	0.291	0.314
	OHWS	0.40	0.59	0.30				
*Patescibacteria*	OH	1.73	1.45	2.79	0.66	0.671	0.331	0.686
	OHWS	1.27	1.88	2.12				
*Actinobacteriota*	OH	5.20	0.64	0.38	1.32	0.556	0.092	0.189
	OHWS	1.73	2.01	0.55				
*Proteobacteria*	OH	0.84	3.05	0.88	0.92	0.163	0.448	0.364
	OHWS	0.47	0.41	0.61				
Stage 2 (120 d)								
*Firmicutes*	OH	44.31 ^b^	61.95 ^a^	54.79 ^a^	4.13	0.039	0.213	0.036
	OHWS	65.31	61.63	56.68				
*Bacteroidota*	OH	48.79 ^a^	31.32 ^b^	38.8 ^ab^	4.54	0.091	0.267	0.047
	OHWS	28.05	32.49	38.50				
*Actinobacteriota*	OH	3.11	3.58	1.39	1.38	0.627	0.628	0.675
	OHWS	1.52	2.69	2.20				
*Patescibacteria*	OH	1.09	1.00	2.41	0.67	0.507	0.463	0.168
	OHWS	2.13 ^a^	0.72 ^b^	0.77 ^b^				
*Spirochaetota*	OH	0.89	0.28	1.55	0.51	0.261	0.909	0.140
	OHWS	1.47	1.77	0.92				

OH, the rations with the forage type of oaten hay; OHWS, the rations with the forage type of oaten hay plus wheat silage; ^a,b^ means with different superscripts were significantly different (*p* < 0.05).

**Table 4:** Microbial community analysis at the genus level (relative abundance > 5%) of microbiomes obtained from rumen fluids of lambs at different feeding stages.

Items	Forage	GAA Addition			SEM	*p*-Value		
		Control	UGAA	CGAA		Forage	GAA	Forage × GAA
Stage 1 (60 d)								
*Ruminococcus*	OH	6.77	28.54	15.75	8.73	0.682	0.286	0.236
	OHWS	13.94	14.39	31.64				
*Prevotella*	OH	12.39 ^b^	9.69 ^b^	26.86 ^a^	4.22	0.313	0.134	0.108
	OHWS	13.10	12.72	12.39				
*Rikenellaceae_RC9_gut_group*	OH	7.87	1.93	5.35	2.60	0.583	0.761	0.104
	OHWS	5.04	9.89	3.79				
*NK4A214_group*	OH	6.02 ^a^	2.29 ^b^	4.23 ^ab^	0.97	0.999	0.269	0.040
	OHWS	4.20	5.39	2.96				
*CAG-352*	OH	0.06	2.38	5.26	3.78	0.395	0.404	0.061
	OHWS	13.71	1.55	0.54				
*Christensenellaceae_R-7_group*	OH	5.09	2.17	3.12	1.08	0.489	0.107	0.095
	OHWS	4.75	5.64	1.87				
*Succiniclasticum*	OH	8.50 ^a^	0.46 ^b^	3.10 ^ab^	1.89	0.368	0.049	0.246
	OHWS	3.87	2.40	1.53				
*Fretibacterium*	OH	0.22	14.93	0.42	5.26	0.279	0.287	0.314
	OHWS	0.32	0.59	0.27				
*norank_f__norank_o__Clostridia_UCG-014*	OH	1.75 ^a^	0.25 ^b^	0.87 ^ab^	3.14	0.189	0.521	0.522
	OHWS	2.77	2.04	8.57				
Stage 2 (120 d)								
*Ruminococcus*	OH	5.14	16.03	12.43	4.76	0.502	0.770	0.027
	OHWS	23.85 ^a^	7.74 ^b^	10.00 ^ab^				
*Prevotella*	OH	30.06 ^a^	12.19 ^b^	11.06 ^b^	4.15	0.214	0.075	0.042
	OHWS	12.78	11.86	15.58				
*Rikenellaceae_RC9_gut_group*	OH	6.58	7.76	15.04	2.97	0.424	0.089	0.769
	OHWS	6.17	6.69	10.58				
*Christensenellaceae_R-7_group*	OH	3.29	7.46	8.47	1.45	0.584	0.119	0.303
	OHWS	5.29	5.74	6.20				
*NK4A214_group*	OH	3.49	4.05	5.43	1.02	0.008	0.040	0.578
	OHWS	4.74	7.25	8.46				
*F082*	OH	2.03	6.91	5.04	1.52	0.358	0.275	0.315
	OHWS	3.34	3.45	3.67				
*Lachnospiraceae_NK3A20_group*	OH	1.00	3.61	1.18	1.16	0.038	0.105	0.966
	OHWS	3.23	5.39	3.54				
*Succiniclasticum*	OH	2.93 ^a^	0.97 ^b^	2.17 ^a^	1.05	0.062	0.226	0.006
	OHWS	1.29	6.88	2.99				
*norank_f__norank_o__Clostridia_UCG-014*	OH	2.31	1.25	2.20	1.36	0.171	0.490	0.170
	OHWS	1.31	5.61	3.58				

OH, the rations with the forage type of oaten hay; OHWS, the rations with the forage type of oaten hay plus wheat silage; ^a,b^ means with different superscripts were significantly different (*p* < 0.05).

**Table 5:** Effects of forage type and GAA addition on ruminal antioxidant capacity.

Items	Forage	GAA Addition			SEM	*p*-Value		
		Control	UGAA	CGAA		Forage	GAA	Forage × GAA
Stage 1 (60 d)								
T-AOC, U/mL	OH	11.29 ^b^	11.91 ^b^	12.75 ^a^	0.25	0.596	<0.001	0.274
	OHWS	11.35 ^b^	12.46 ^a^	12.48 ^a^				
SOD, U/mL	OH	97.07 ^c^	123.57 ^b^	176.26 ^a^	2.54	0.061	<0.001	<0.001
	OHWS	96.79 ^c^	184.66 ^a^	127.49 ^b^				
CAT, U/mL	OH	8.60 ^b^	8.97 ^b^	10.06 ^a^	0.21	0.259	<0.001	<0.001
	OHWS	8.45 ^c^	10.31 ^a^	9.46 ^b^				
GSH-Px, U/mL	OH	806.07 ^c^	840.07 ^b^	924.25 ^a^	8.78	0.898	<0.001	<0.001
	OHWS	800.20 ^c^	924.27 ^a^	848.73 ^b^				
GSH, nmol/mL	OH	9.23 ^b^	9.69 ^b^	10.84 ^a^	0.21	0.123	<0.001	0.060
	OHWS	9.32 ^b^	10.55 ^a^	10.71 ^a^				
MDA, nmol/mL	OH	5.82 ^a^	5.33 ^a^	5.20 ^b^	0.17	0.305	<0.001	0.274
	OHWS	5.84 ^a^	4.87 ^b^	5.21 ^b^				
Stage 2 (120 d)								
T-AOC, U/mL	OH	13.87 ^c^	14.60 ^b^	15.22 ^a^	0.19	0.097	<0.001	0.001
	OHWS	14.12 ^c^	15.61 ^a^	14.75 ^b^				
SOD, U/mL	OH	107.31 ^b^	121.5 ^b^	169.68 ^a^	5.35	0.179	<0.001	<0.001
	OHWS	95.68 ^c^	183.83 ^a^	137 ^b^				
CAT, U/mL	OH	10.46	10.75	11.00	0.40	0.322	0.048	0.110
	OHWS	10.32 ^b^	12.08 ^a^	10.81 ^ab^				
GSH-Px, U/mL	OH	931.47 ^b^	948.30 ^b^	1007.26 ^a^	12.54	0.619	<0.001	<0.001
	OHWS	909.69 ^c^	1031.11 ^a^	961.74 ^b^				
GSH, nmol/mL	OH	11.81	12.16	12.63	0.26	0.370	0.009	0.845
	OHWS	11.50 ^b^	12.12 ^ab^	12.40 ^a^				
MDA, nmol/mL	OH	6.15 ^a^	6.16 ^a^	5.30 ^b^	0.12	0.454	<0.001	<0.001
	OHWS	6.29 ^a^	5.48 ^b^	5.61 ^b^				

OH, the rations with the forage type of oaten hay; OHWS, the rations with the forage type of oaten hay plus wheat silage; T-AOC, total antioxidant capacity; SOD, superoxide dismutase; CAT, catalase; GSH-Px, glutathione peroxidase; GSH, glutathione; MDA, malondialdehyde; S.E.M, standard error of least squares means; ^a,b,c^ means with different superscripts were significantly different (*p* < 0.05).

## Data Availability

All data relevant to the study are included in the article or uploaded as supplementary information. Data are available on reasonable request.

## Supplementary Materials

The following supporting information can be downloaded at: https://www.mdpi.com/article/10.3390/antiox12030772/s1, Table S1: Effects of forage type and GAA addition on alpha diversity of ruminal microbiota.

## Appendix A

**Table A1:** Ingredient and chemical composition of trial diets.

Ingredients	Stage 1		Stage 2	
	OH	OHWS	OH	OHWS
Wheat silage ^1^	0	90	0	70
Oat hay ^2^	250	160	200	130
Concentrate ^3^	750	750	800	800
Nutrient level of TMR (g/kg, as Dry Matter)				
Organic matter	893.3	901.8	905.6	910.5
Crude protein	191.9	193.7	184.6	174.5
Ether extract	36.6	35.1	38.0	37.5
Neutral detergent fiber	307.6	293.8	274.5	271.2
Acid detergent fiber	118.4	117.1	108.0	99.7
Net energy for gain (MJ/kg)	4.60	4.64	4.79	4.81

OH, the rations with the forage type of oaten hay; OHWS, the rations with the forage type of oaten hay plus wheat silage. Wheat silage ^1^: DM 329.3 g/kg; CP 120.8 g/kg; EE 32.0 g/kg; NDF 620.0 g/kg; ADF 370.5 g/kg; OM 919.0 g/kg; NE_g_ 2.51 MJ/kg. Oat hay ^2^: DM 879.5 g/kg; CP 121.0 g/kg; EE 24.1 g/kg; NDF: 611.6 g/kg; ADF: 351.4 g/kg; OM 917.0 g/kg; NE_g_ 1.67 MJ/kg. Concentrate ^3^: Contained per kg in stage 1: 380 g Corn meal, 150 g Soybean meal, 180 g DDGS, 40 g Premix, Contained per kg in stage 2: 500 g Corn meal, 150 g Soybean meal, 110 g DDGS, 40 g Premix. Contained per kg premix: 200–500 mg Cu, 750–17,500 mg, 750–5000 mg Mn, 1250–4250 mg Zn, 3.75–350 mg I, 2.5–17.87 mg Se, 75,000–350,000 IU vitamin A, 12,500–142,500 IU vitamin D3, 500 mg vitamin E.

**Table A2:** PCR amplification of 16S rRNA gene.

Stages	Temperature	Time	Cycles
Initial denaturation	95 °C	3 min	1
Denaturation	95 °C	30 s	27
Annealing	55 °C	30 s	1
Extension	72 °C	45 s	1
Single extension	72 °C	10 min	1

**Table A3:** PCR system.

Items	Volume
5 × FastPfu Buffer	4 μL
2.5 mmol/L dNTPs	2 μL
Forward primer (5 mmol/L)	0.8 μL
Reverse primer (5 mmol/L)	0.8 μL
FastPfu Polymerase	0.4 μL
Template DNA	10 ng
ddH_2_O	12 μL
Total	20 μL
