# Supplementary material for: Dietary Guanidine Acetic Acid Improves Ruminal Antioxidant Capacity and Alters Rumen Fermentation and Microflora in Rapid-Growing Lambs
Source: Antioxidants (Basel). 2023 Mar 22;12(3):772. doi: 10.3390/antiox12030772 (PMC10044800; doi:10.3390/antiox12030772)
Supplement: Supplementary file 1 [file antioxidants-12-00772-s001.zip › antioxidants-2240279-supplementary.pdf]

Table S1. Effects of forage type and GAA addition on alpha diversity of ruminal microbiota

Items	Forage	GAA addition			SEM	*p*-value		
		**control**	**UGAA**	**CGAA**		**Forage**	**GAA**	**Forage × GAA**
Stage 1 (60 d)								
sobs	OH	803	769	742	77.20	0.837	0.848	0.935
	OHWS	784	806	763				
shannon	OH	4.78	3.96	4.47	0.29	0.968	0.398	0.120
	OHWS	4.49	4.69	4.07				
simpson	OH	0.02	0.09	0.03	0.02	0.974	0.403	0.051
	OHWS	0.04	0.03	0.08				
ace	OH	977.12	969.76	895.42	87.94	0.872	0.856	0.874
	OHWS	949.03	972.14	955.80				
chao	OH	983.17	972.26	890.65	82.72	0.795	0.788	0.836
	OHWS	952.68	987.24	959.66				
Stage 2 (120 d)								
sobs	OH	759	661	691	33.20	0.017	0.249	0.431
	OHWS	780	763	783				
shannon	OH	4.57	4.42	4.5	0.17	0.112	0.741	0.355
	OHWS	4.52	4.81	4.81				
simpson	OH	0.03	0.03	0.03	0.01	0.833	0.302	0.194
	OHWS	0.05	0.02	0.03				
ace	OH	926.20	824.50	849.65	35.77	0.011	0.152	0.661
	OHWS	973.77	936.91	939.90				
chao	OH	928.79	834.09	849.33	39.92	0.010	0.245	0.729
	OHWS	985.44	949.49	957.45				

OH, the rations with the forage type of oaten hay; OHWS, the rations with the forage type of oaten hay plus wheat silage.
